# The Effect of Glass Structure on the Luminescence Spectra of Sm^3+^-Doped Aluminosilicate Glasses

**DOI:** 10.3390/ma16020564

**Published:** 2023-01-06

**Authors:** Andreas Herrmann, Mohamed Zekri, Ramzi Maalej, Christian Rüssel

**Affiliations:** 1 Department of Inorganic-Nonmetallic Materials, Institute of Materials Science and Engineering, Ilmenau University of Technology, Gustav-Kirchhoff-Str. 5, 98693 Ilmenau, Germany; 2 LaMaCoP, Faculty of Sciences of Sfax, Sfax University, Sfax 3018, Tunisia; 3 Otto Schott Institute of Materials Research, Jena University, Fraunhoferstr. 6, 07743 Jena, Germany

**Keywords:** aluminosilicate glass, glass structure, hypersensitivity, rare earth site symmetry, samarium, molecular dynamics simulations, luminescence

## Abstract

Peralkaline Sm^3+^-doped aluminosilicate glasses with different network modifier ions (Mg^2+^, Ca^2+^, Sr^2+^, Ba^2+^, Zn^2+^) were investigated to clarify the effect of glass composition and glass structure on the optical properties of the doped Sm^3+^ ions. For this purpose, the Sm^3+^ luminescence emission spectra were correlated with the molecular structure of the glasses derived by molecular dynamics (MD) simulations. The different network modifier ions have a clear and systematic effect on the peak area ratio of the Sm^3+^ emission peaks which correlates with the average rare earth site symmetry in the glasses. The highest site symmetry is found for the calcium aluminosilicate glass. Glasses with network modifier ions of lower and higher ionic radii show a notably lower average site symmetry. The symmetry could be correlated to the rare earth coordination number with oxygen atoms derived by MD simulations. A coordination number of 6 seems to offer the highest average site symmetry. Higher rare earth coordination probabilities with non-bridging oxygen result in an increased splitting of the emission peaks and a notable broadening of the peaks. The zinc containing glass seems to play a special role. The Zn^2+^ ions notably modify the glass structure and especially the rare earth coordination in comparison to the other network modifier ions in the other investigated glasses. The knowledge on how glass structure affects the optical properties of doped rare earth ions can be used to tailor the rare earth absorption and emission spectra for specific applications.

## 1. Introduction

Rare earth-doped glasses are the backbone of today’s telecommunication. Erbium (Er^3+^)-doped silica-based glass fibers are used for amplification of optical signals in long distance fiberoptic cables which currently span the whole globe and make high-speed Internet, world-wide broadcasting, and communication possible. However, rare earth-doped glasses are also used as optical amplification medium for lasers, or can be used as optical sensors or wavelength converters for lighting and solar applications. Crucial for the use of rare earth-doped materials are their optical properties, which strongly depend on the host material. Crystalline materials are, in this respect, well investigated and understood; however, the effect of glass composition and molecular structure on the properties of the doped rare earth ions is not yet fully decoded. The main reasons are the amorphous, irregular molecular structure of glasses and the low rare earth doping concentrations that usually do not exceed about 1 mol%.

In our previous publications [1,2], among others, we tried to clarify the effect of glass composition and glass structure of aluminosilicate glasses on the molecular level on the optical properties of doped rare earth ions. Sm^3+^- and Er^3+^-doped glasses and their optical properties were respectively investigated in these publications. Aluminosilicate (AS) glasses offer some advantages in comparison to other glass types, e.g., in many cases better mechanical properties and lower coefficients of thermal expansion, and also notably broadened rare earth absorption and emission spectra [3,4]. All these properties are beneficial for their potential application as laser-active material, especially for generation of high-power laser pulses; however, their mechanical properties also strongly depend on their actual composition.

In principle, only a few methods are suitable to study the incorporation of rare earth elements into glasses. Raman and infrared spectroscopy are not sensitive enough for taking the small rare earth concentration usually present in luminescent glasses into account. Despite the paramagnetism of rare earth ions, Electron Paramagnetic Resonance (EPR) spectroscopy does not provide useful information (with the exception of Eu^2+^) and Nuclear Magnetic Resonance (NMR) spectroscopy is strongly affected by the strong paramagnetic shift induced by the rare earth elements. Mößbauer spectroscopy can only be applied for europium (^151^Eu) due to the lack of other suitable radionuclides. A remaining favorable method seems to be extended X-ray absorption fine structure (EXAFS) which, however, requires synchrotron radiation and hence is not widely available. The only favorable method to gain insight into the incorporation of rare earth ions to the glass structure at low doping concentrations is the method of molecular dynamic (MD) simulations, which gives the actual three-dimensional atomic structure of the glasses, including the rare earth dopants’ coordination with neighboring atoms. By using this method, we were so far able to correlate the increased splitting of absorption and emission peaks to increased coordination numbers of the doped rare earth ions with so called non-bridging oxygen (NBO) atoms ^-^O–Si≡, i.e., oxygen atoms that are not bond to two network former ions (e.g., ≡Si–O–(Al, Si)≡) and therefore represent a “break” in the glass network [2,5,6]. On the other hand, we proposed a correlation of the overall average rare earth coordination number with the rare earth site symmetry and the intensity of the so called “hypersensitive” optical rare earth transitions in the investigated glasses [2]. These absorption or emission transitions show a much higher sensitivity in their oscillator strengths, i.e., their intensity, than other non-hypersensitive transitions to changes of the host material or even single ligands [7,8]. In fact, the correlation between rare earth site symmetry and intensity of the hypersensitive transitions is already proven in crystalline materials [7]; however, up to now, it is unclear, how exactly the rare earth site symmetry is affected in glasses. In the following, we report on luminescence spectra of Sm^3+^-doped alkaline earth aluminosilicate glasses and attributed MD simulations. Previous results from luminescence spectroscopy [1] and MD simulations [2] are revisited and included in the discussion. In comparison to alkali AS glasses, alkaline earth-containing AS glasses show lower melting temperatures and are therefore easier to produce and possess in most cases better mechanical properties [3].

For a short or a more comprehensive introduction to aluminosilicate glass structure, the reader is referred to [1] and [9], respectively.

## 2. Materials and Methods

*Glass preparation:* For each glass composition, 100 to 200 g of glass was prepared by using oxides and carbonates of the molar compositions given in Table 1. The used raw materials were MgO (Merck KG, Germany), CaCO_3_ (Merck KG, Germany), SrCO_3_ (Reachim, Ukraine), BaCO_3_ (Reachim, Ukraine), ZnO (Merck KG, Germany), Al_2_O_3_ (Pengda Munich GmbH, Germany), SiO_2_ (Sipur A1, Schott Jenaer Glas GmbH, Germany), and Sm_2_O_3_ (Auer-Remy GmbH, Germany). All glasses are peralkaline aluminosilicate glasses with a molar ratio of 35/10/55 of network modifier oxide, Al_2_O_3_, and SiO_2_, respectively. Consequently, the samples are denoted as XAS3510, where X stands for the used network modifying ion. The doping concentration of Sm^3+^ was kept constant at 1×10^20^ ions/cm^3^. This corresponds to about 0.2 mol% Sm_2_O_3_, depending on the density of the glass. A constant rare earth volume concentration is important to avoid different luminescence concentration quenching intensities for different glass samples. The batches were thoroughly mixed, transferred to a platinum crucible, and then placed in an electric furnace for melting between 1550 and 1630 °C, depending on glass composition. To assure a good glass quality, the melt was shaken from time to time. Usually, the melting time was between 2 and 3 h. Afterwards, the glass melts were removed from the furnace, poured on a brass block, and transferred to a cooling furnace preheated to around 20 K above the glass transition temperature T_g_. Subsequently, the cooling furnace was switched off, which allowed the samples to cool down gradually over night. In this way, homogeneous and bubble-free glasses were obtained. From the glass blocks, samples of 1 cm thickness were cut and polished for the optical measurements.

*Glass characterization:* The glass densities were measured using an AccPyc 1330 pycnometer (Micromeritics GmbH, Unterschleißheim, Germany). The error of these measurements was lower than ±0.03 g/cm^3^. The transition temperatures were derived by using a differential scanning calorimeter (DSC PT 1600, Linseis GmbH, Selb, Germany) at a heating rate of 10 K/min. For the determination of the exact transition temperature, T_g_, the onset point of the DSC curve was used. The error of this method is estimated to about ±5 K. 

The refractive indices were measured using a Pulfrich-Refractometer PR2 (VEB Carl Zeiss Jena, Jena, Germany). The measurement error depends strongly on the optical quality of the glasses and is about ±0.002. The luminescence emission and excitation spectra were recorded using a luminescence spectrometer RF-5301PC (Shimadzu, Kyoto, Japan) operated in reflection mode.

The optical basicities Λ of the glasses were calculated from their molar compositions according to Duffy [10] using the averaged basicity values of Duffy and Lebouteiller/Courtine published in [11]. The used values are Λ_SiO_2__ = 0.48, Λ_Al_2_O_3__ = 0.603, Λ_MgO_ = 0.78, Λ_ZnO_ = 0.935, Λ_CaO_ = 1.00, Λ_SrO_ = 1.10, and Λ_BaO_ = 1.15.

*Molecular dynamic simulations:* The molecular dynamic (MD) simulations were conducted as stated in detail in our previous publications on the topic [5,12]. We used a specific method of MD simulations, the so-called “inherent structure (IS) sampling“. This method uses relatively small sets of atoms (around 400 in our case) but simulates a large number of them (3000 in our case). The molecular structures of these 3000 IS are then statistically analyzed by weighting them by their potential energy per atom. That means an inherent structure of relatively high potential energy is regarded with less weight than a structure with comparably low potential energy, since a low energy structure is assumed to better represent the real atomic structure of the glass. With this method, we obtained the coordination of 6000 rare earth ions for each of the different glass compositions. At a doping concentration of about 0.5 at% (two rare earth atoms per 400 atoms in each IS), a MD simulation of about 1,200,000 atoms with the conventional method would be needed, which would probably take years to be calculated with recent computational power. In [12], we have also shown that the inherent structure sampling results in similar molecular structures as the conventional MD simulation method (here compared to a simulation size of 6460 atoms). 

For the MD simulations, the Large-Scale Atomic/Molecular Massively Parallel Simulator (LAMMPS, Sandia National Laboratories, Albuquerque, NM, USA) [13] along with the empirically parameterized interatomic potential functions of Pedone et al. [14] were employed. This parametrization already proved to accurately predict the atomic structure of various silica-based glasses [15,16,17,18]. Computation of short-range interactions used a cutoff of 15 Å, and for summation of long-range Coulomb interactions, the particle-particle-particle-mesh method [19] was applied. Integration of the equations of motion used the velocity Verlet algorithm [20] with a time step of 1 fs. At first, initial structures were generated by randomly placing ions according to the molar glass composition (Gd_2_NM_50_Al_29_Si_79_O_253_, Σ = 413 atoms, NM = network modifier atom) into a cubic unit cell with a volume corresponding to the experimentally observed mass density of the glass composition [1]. Next, the initial structures were geometrically optimized and equilibrated at T = 3000 K for 6.5 ns using the canonical (NVT, constant particle number N, volume V and temperature T) ensemble along with the Nosé-Hoover thermostat [21,22] and employing periodic boundary conditions. During the last 6 ns, geometry optimizations under constant (zero) pressure conditions were applied to structures taken from the MD trajectory every 2 ps yielding the 3000 inherent structures for each glass composition. In the simulations Gd^3+^ was chosen as model rare-earth ion because there are no potential functions published so far for Sm^3+^, and because Gd^3+^ has a similar ionic radius as Sm^3+^ (R_Gd_ = 0.94 Å, R_Sm_ = 0.96 Å) [23].

## 3. Results and Discussion

### 3.1. Peak Area Ratio and Sm^3+^ Site Symmetry

**Figure 1:**
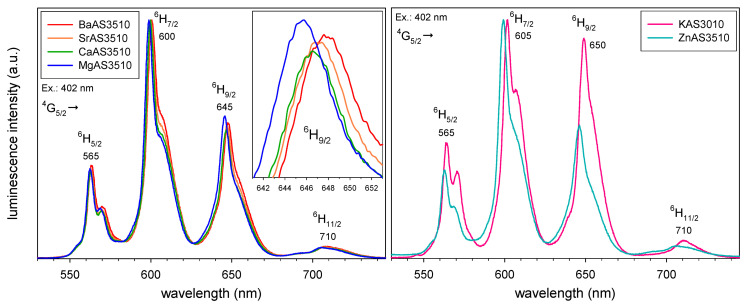
Sm^3+^ emission spectra of different alkaline earth aluminosilicate glasses (**left**), and of a potassium and a zinc aluminosilicate glass (**right**) excited at around 402 nm. The spectra are normalized to their most intense peak at around 600 nm. The peak intensity of the peak at 645 nm shows clear differences in dependence of the host glass (magnified in the inset left).

The left diagram in Figure 1 shows the emission spectra of Sm^3+^-doped peralkaline aluminosilicate glasses with different network modifier (NM) ions, Mg^2+^, Ca^2+^, Sr^2+^, and Ba^2+^. The doping concentration is constant at 1 × 10^20^ Sm^3+^ ions/cm^3^. The sample data have already been published in [1] and the spectrum of sample BaAS3510 has already been shown there; however, here the spectra of different samples are added for comparison. Sm^3+^ shows a strong orange-red luminescence emission if excited in the UV-blue spectral region. The typical Sm^3+^ emission spectrum consists of four distinct emission peaks at around 565, 600, 645, and 710 nm due the transitions from the energy level ^4^G_5/2_ to the levels ^6^H_5/2_, ^6^H_7/2_, ^6^H_9/2_, and ^6^H_11/2_, respectively [24,25]. The spectra in Figure 1 are normalized to their highest emission peaks at around 600 nm. As shown in Figure 1 (left), the relative intensity of the peak at 645 nm changes with glass composition and shows a minimum for the CaAS3510 composition. For more clarity, the spectral region around this peak is shown in more detail in the inset of Figure 1 (left). The transition ^4^G_5/2_→^6^H_9/2_ at about 645 nm has ΔJ = 2 and therefore is a pure electric dipole transition [25]. Although typically not considered as a hypersensitive transition, it is heavily affected by the electrical field surrounding the Sm^3+^ ion. The transition ^4^G_5/2_→^6^H_7/2_ at about 600 nm has ΔJ = 1 and a notable magnetic dipole contribution [25]. Therefore, this transition is less affected by changes in the local electrical field. As already shown in [1] and elsewhere (e.g., [26,27,28,29]), the ratio of these two Sm^3+^ emission peaks is highly sensitive to structural changes in the host composition. For this reason, the peak area ratio A_645_/A_600_ of the peaks at 645 nm and 600 nm was calculated by integrating the peaks in energy scale. These data are added to Table 1. The peak area ratio A_645_/A_600_ corresponds to the asymmetry at the Sm^3+^ site. That means the Sm^3+^ ions in the glass CaAS3510 on average show the lowest asymmetry (=highest symmetry) of the investigated glasses. The glass compositions with NM ions of smaller (Mg^2+^) and larger ionic radii (Sr^2+^, Ba^2+^) show a lower average symmetry at the Sm^3+^ sites. It must be noted that the differences in the peak area ratios of the glasses shown here are comparably small. With different network modifying ions, most notably NM ions of low field strength (e.g., alkali ions), and lower NM/Al_2_O_3_ ratios, the Sm^3+^ emission spectra can be altered more fundamentally. As an example, the emission spectrum of the Sm^3+^-doped peralkaline potassium aluminosilicate glass KAS3010 from [1] is shown in the right diagram of Figure 1. The peak at 645 nm can be even the most intense, thereby changing the overall emission color to a deeper red [1,27,29]. This shows how specific rare earth emission or absorption peaks can be amplified or attenuated by changing the host glass composition.

**Figure 2:**
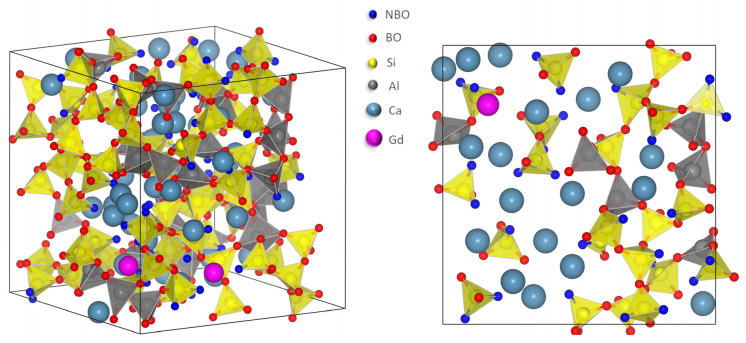
Molecular dynamic simulation results. **Left picture:** An inherent structure of 413 atoms of the glass composition CaAS3510 as obtained by the MD simulations. **Right picture:** A slice of the CaAS3510 composition showing its structure in more detail. SiO_4_ tetrahedra (yellow) and [AlO_4_]^-^ tetrahedra (gray) interconnected by bridging oxygen (BO, red) form the three-dimensional glass network. The network modifying ions Ca^2+^ (light blue) and Gd^3+^ (pink) interrupt the network and form depolymerized regions. Their charge is compensated by non-bridging oxygen (NBO, blue) and [AlO_4_]^-^. Note that the structure extends behind and in front of this slice.

The left part of Figure 2 shows a glass structure as obtained by the MD simulations, actually one of 3000 inherent structures of the CaAS3510 glass composition. For more clarity, a slice of the three-dimensional structure has been cut out (right picture in Figure 2). Clearly to be seen are the interconnected SiO_4_ (yellow) and [AlO_4_]^-^ tetrahedra (gray) which form the three-dimensional glass network. The tetrahedra are connected by bridging oxygen (BO, red). Note that the structure continues in front and behind the shown slice, which is noted by the red BO that are not connected in the picture. The network is interrupted by the network modifying (NM) ions Ca^2+^ (light blue) and Gd^3+^ (pink). Their charge is compensated by non-bridging oxygen (NBO, blue) and [AlO_4_]^-^ groups.

Table 2 shows the coordination numbers (CN) and distances of the doped rare earth ions, Gd^3+^ in this case, with their neighboring atoms in the first (O) and second (Si, Al, NM) coordination sphere derived from the statistical analysis of the MD simulation results. The results of the barium- and magnesium-containing compositions are taken from [2]. The coordination numbers with oxygen systematically decrease with increasing ionic radius of the NM ion, from about 6.06 for MgAS3510 to about 5.60 for the BaAS3510 glass. Additionally, the glass composition ZnAS3510 was investigated, since Zn^2+^ has a similar ionic radius as Mg^2+^ (R_Zn_ = 0.60 Å, R_Mg_ = 0.57 Å, both at CN 4 [23]) and shows a peak area ratio between MgAS3510 and CaAS3510 [1]. Therefore, also a similar glass structure as for MgAS3510 was assumed. However, as the simulations show, the Gd^3+^ coordination number in ZnAS3510 (5.54) is much different from MgAS3510 (6.06) and even slightly smaller than for BaAS3510 (5.60) (Table 2). This shows that the Zn^2+^ ion has a different effect on the glass structure than the alkaline earth ions, despite its similar emission spectrum. For comparison, the emission spectrum of Sm^3+^-doped ZnAS3510 is added to the right diagram in Figure 1. As already stated, it is very similar to the spectrum of MgAS3510.

Although the MD simulations were conducted with Gd^3+^ as a model rare earth ion, the simulation results can be compared to the spectral measurements of Sm^3+^-doped samples. It is assumed that both rare earth ions are incorporated to the glass structure in a similar way. However, Gd^3+^ has a slightly smaller ionic radius (R_Gd_ = 0.94 Å) than Sm^3+^ (R_Sm_ = 0.96 Å) (both at a CN of 6) [23]. That means, the actual coordination numbers of Sm^3+^ are probably even slightly higher than the values obtained for Gd^3+^.

**Figure 3:**
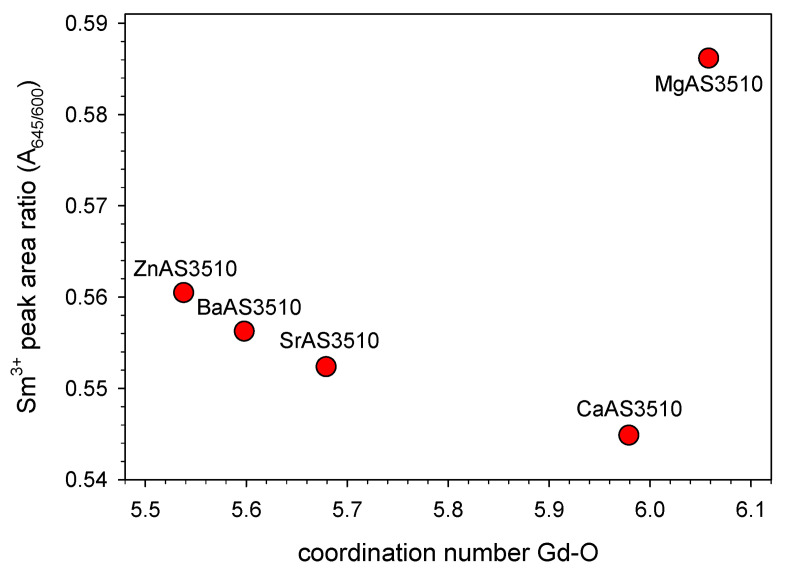
Sm^3+^ emission peak area ratio of the peaks at 645 and 600 nm in dependence of the rare earth (Gd^3+^) coordination number with oxygen from molecular dynamics simulations for the investigated aluminosilicate glasses. The lowest peak area ratio corresponds to the highest average symmetry at the rare earth sites in the glass structure, which is found at a coordination number of around 6.

In Figure 3, the peak area ratios A_645_/A_600_ from the emission spectra are plotted versus the Gd^3+^ coordination numbers obtained from the MD simulations. As already discussed above, the sample CaAS3510 with a coordination number close to 6 offers the highest average symmetry for the doped Sm^3+^ ions. Higher coordination numbers in MgAS3510 and lower coordination numbers in SrAS3510, BaAS3510, and ZnAS3510 offer, on average, a lower site symmetry. Interestingly, the data point of ZnAS3510 is perfectly in line with the other measurements, although not close to MgAS3510 as initially assumed, but rather close to BaAS3510. In our previous publication on this matter, we already found an increasing symmetry for increasing coordination numbers from about 5.4 to 6.0 for Er^3+^-doped glasses of similar compositions and assumed, that the coordination number 6 could mark a symmetry maximum [2]. Unfortunately, Er^3+^ has a much smaller ionic radius (R_Er_ = 0.89 Å) than Gd^3+^ and Sm^3+^ [23]. Therefore, no higher coordination numbers than 6 could be generated in these glasses and the assumed maximum symmetry at a CN of 6 could not be proven. The larger Sm^3+^ ion, on the other hand, shows a higher coordination number than 6 in MgAS3510 and, as shown in Figure 2, its site symmetry in this glass is much lower than for the other samples. However, it is only one of five data points. From a geometrical point of view, a maximum average symmetry at a coordination of 6 is reasonable. A typical coordination could for instance be a (distorted) octaeder with the rare earth ion in the center.

The zinc aluminosilicate glass seems to play a special role. Despite the relatively small ionic radius of Zn^2+^, the rare earth ions in this sample have a comparably low coordination number. The reason for this behavior could be the ZnO’s character as a so-called “intermediate” oxide, i.e., an oxide with characteristics between typical network formers, such as SiO_2_, and typical network modifiers, such as the alkaline earth oxides. In Table 3, the coordination of the network modifier ions in their first and second coordination sphere derived from the MD simulations are compared. Interestingly, Zn^2+^ shows by far the smallest coordination number with NM ions in the glass despite its higher ionic radius than Mg^2+^. Zn^2+^ seems to be integrated into the glass forming network of SiO_4_ and [AlO_4_]^-^ tetrahedra, preferably forming ZnO_4_ tetrahedra itself. This can be assumed from its low CN with oxygen, which is only 4.2, i.e., much lower than for all other NM ions investigated here, e.g., 4.6 for Mg^2+^ (Table 3). Moreover, its bond length of 1.95 Å to neighboring oxygen atoms is the lowest of all divalent cations (Table 3). In [30], Cormier and coworkers also suggest that Zn competes with aluminum in network-forming positions. Therefore, its tendency to coordinate with other network modifying ions in the glass structure is lower. Consequently, the rare earth ions in this glass face a molecular structure which is less modified and rather resembles a metaluminous composition, i.e., a composition where formally all network modifier charges are compensated by [AlO_4_]^-^ groups and where the structure is dominated by a higher polymerization, i.e., a higher percentage of ≡Si–O–(Al, Si)≡ chains. As reported in [5,12], metaluminous aluminosilicate glasses generally show notably lower rare earth CN than peralkaline ones, which fits to the above explanations. Even the comparably low T_g_ and melting temperature of ZnAS glasses can probably be explained by this structural model. The ≡Si–O–(Al, Si)≡ chains are probably weakened by frequent incorporation of ZnO_4_ tetrahedra. However, these assumptions must be discussed in more detail in future works.

According to the previous statements on rare earth symmetry, the glass composition CaAS3510 should offer the highest average Sm^3+^ symmetry of all glass compositions in [1]. Interestingly, this is not the case. Some of the peralkaline lanthanum aluminosilicate glasses seem to offer an even slightly higher average Sm^3+^ symmetry. This could either be explained by a Sm^3+^ CN closer to 6 in these glasses or by the chemical similarity of La^3+^ and Sm^3+^, resulting in a less disturbed surrounding and a lower variation in the second coordination sphere of the doped Sm^3+^ ions. This shows that the coordination with neighboring atoms in the first coordination sphere is not the only parameter that determines symmetry, but probably the most important one. However, more investigations are needed to clarify the role of the atoms in the second coordination sphere of the rare earth ions.

### 3.2. Peak Position, Optical Basicity, and Peak Broadening

The spectra in Figure 1 do not only give information on Sm^3+^ site symmetry. As clearly seen in the inset of Figure 1 (left), the emission peaks shift to higher wavelengths with increasing ionic radius of the network modifier ion, i.e., the ^4^G_5/2_→^6^H_9/2_ peak maximum is at around 645 nm for the MgAS3510 sample but at about 649 nm for the BaAS3510 sample. For ZnAS3510, it is at 646 nm, i.e., between MgAS3510 and CaAS3510 (Figure 1 (right)). The same effect applies to all other peaks, and even for other NM/Al_2_O_3_ ratios (e.g., [1]). The peak shift is a well-known effect and can be correlated to the optical basicity of the glasses. Higher optical basicity values result in a shift of the emission (and absorption) peaks to longer wavelengths. For this reason, the optical basicity values are added to Table 1.

Another effect seen in Figure 1 is probably more subtle. It can be noted for the emission peaks at 565 and 600 nm. The peaks get broader with increasing ionic radius of the network modifier ion. Since the peak intensities of the 600 nm peaks are normalized, the peak areas A_600_ are showing this effect nicely (Table 1). The A_600_ values increase in the order MgAS3510 < CaAS3510 < SrAS3510 < BaAS3510. The zinc containing glass, again, does not fit, probably due its different structure, as discussed above. Another nice example for this effect is the spectrum of KAS3010 in the right diagram of Figure 1. Its 565 and 600 nm peaks are clearly broader than those of all other spectra in Figure 1. There are two main contributions to this effect, firstly the different variation of rare earth sites in the different glass samples (inhomogeneous broadening) and secondly an increased peak splitting due to an increased electrical field strength at the rare earth positions in the glass network. The second usually also results in a more subdivided peak structure, i.e., additional and more pronounced subpeaks become visible, which is not the case for the glass samples investigated here. However, it can nicely be observed for Er^3+^ [2,31] and Tb^3+^ [6] doped glasses of similar compositions. In our previous publications [2,5], we could clearly correlate the increased peak splitting to an increased coordination of the rare earth ions with non-bridging oxygen (NBO), which have a strongly localized negative charge and therefore expose the neighboring rare earth ion to a stronger electrical field than neighboring bridging oxygen atoms would do. As seen in Table 2, the Gd^3+^ coordination numbers with NBO increase from MgAS3510 (4.05) to BaAS3510 (4.28). The rare earth coordination number with NBO is correlated to the field strength difference between rare earth ion and network modifier ion, since the rare earth ions compete with the NM ions for network positions that can compensate their own charge and have a high local field strength, i.e., the rare earth ions prefer positions with sufficiently high coordination numbers with NBO sites [2,5]. If the NM ions have a high field strength (= small ionic radius), as e.g., Mg^2+^, the rare earth ions are forced into less preferred positions for charge compensation, probably close to [AlO_4_]^-^ groups with a lower local field strength. Consequently, the Gd^3+^ coordination number with Al in the second coordination sphere is highest in the MgAS3510 and lowest for the BaAS3510 sample (Table 2).

The different variation of rare earth sites in the different glass samples of constant NM/Al_2_O_3_ ratio is less easy to explain. It is well known that introduction of Al_2_O_3_ to silicate glasses reduces the rare earth peak splitting, but increases the inhomogeneous broadening of the rare earth spectra (e.g., [32,33,34]). Al_2_O_3_ is mostly incorporated in the silicate glass structure as [AlO_4_]^-^ groups (at NM/Al_2_O_3_ ratios close to or lower than 1, nevertheless also minor quantities of five- and six-fold coordinated Al occur) [5,9,12]. Because of the negative charge of the [AlO_4_]^-^ group, the Al_2_O_3_ addition decreases the coordination of the rare earth ions with NBO and, due to the addition of an additional coordination partner, i.e., different from SiO_4_ tetrahedra, increases the variation of sites. The effect of the NM ions is much smaller and more difficult to determine. For the samples investigated here, the peak splitting is probably the determining factor for the peak broadening, i.e., the subpeaks (shoulders) at the long wavelength side of the Sm^3+^ emission peaks at 565 and 600 nm are split further apart from the main peak and therefore increase the overall peak width. Again, this effect is most obvious for the KAS3010 glass (Figure 1 (right)).

## 4. Conclusions

By using molecular dynamic (MD) simulations in connection with measurements of the luminescence emission spectra of Sm^3+^-doped aluminosilicate glasses with different compositions, the effect of different network modifier ions on the glass structure, the local rare earth sites and their emission spectra could be clarified. Coordination of the rare earth ions with an increasing number of non-bridging oxygen atoms increases the peak splitting of the emission peaks and therefore increases the width of the peaks. The overall coordination number of the rare earth ions with oxygen atoms correlates with the rare earth site symmetry in the glass structure. Here, an average coordination number of 6 seems to mark a maximum in the site symmetry. Lower and higher average rare earth coordination numbers result in a lower average rare earth site symmetry. This affects the emission (and absorption) intensities of the so-called hypersensitive peaks.

The MD simulations also show that Zn^2+^ ions are preferably integrated into the glass forming network as ZnO_4_ tetrahedra. Therefore, the glass network and rare earth coordination are notably modified in comparison to the alkaline earth containing glasses. In the zinc containing aluminosilicate glass the rare earth ions have unexpectedly low coordination numbers and a relatively low site symmetry.

The knowledge on how glass composition actually affects the optical properties of doped rare earth ions allows to tailor the rare earth spectra for specific applications, i.e., to amplify or attenuate specific (laser) peaks and thereby to increase the laser efficiency, change the color of the rare earth emission, or broaden the emission peaks for an improved compression of rare earth generated laser pulses.

## Figures and Tables

**Table 1:** Chemical compositions, densities, transition temperatures T_g_, refractive indices n_e_, theoretical optical basicities Λ, peak areas A_645_, A_600_ and peak area ratios A_645_/A_600_ of the transitions at 645 nm (“hypersensitive”) and the transitions at 600 nm of the studied samples.

Sample Name	MgAS3510	ZnAS3510	CaAS3510	SrAS3510	BaAS3510
Network modifier oxide (mol%)	(MgO) 35	(ZnO) 35	(CaO) 35	(SrO) 35	(BaO) 35
Al_2_O_3_ (mol%)	10	10	10	10	10
SiO_2_ (mol%)	55	55	55	55	55
density(g/cm^3^)	2.62	3.24	2.80	3.28	3.75
transition temperature T_g_ (°C)	811	705	812	795	771
refractive index n_e_	1.562	1.614	1.594	1.599	1.621
theoretical optical basicity Λ	0.561	0.592	0.605	0.625	0.635
peak area A_600_ (cm^−1^)	404.140	417.650	404.985	414.462	424.554
peak area A_645_ (cm^−1^)	236.895	234.086	220.666	228.934	236.162
peak area ratioA_645_/A_600_	0.586	0.560	0.545	0.552	0.556

**Table 2:** Gd^3+^ coordination according to molecular dynamic simulations. **Upper part of the table:** the coordination numbers (CN) of Gd^3+^ with different oxygen species in the first coordination sphere in the investigated glasses and the percentages of the different oxygen coordinations. The Gd-O distances are constant at 2.25 Å. NBO: non-bridging oxygen, BO: bridging oxygen, Tri: oxygen triclusters. **Lower part:** the coordination numbers (CN) of Gd^3+^ with different cations Al^3+^, Si^4+^, Mg^2+^, Zn^2+^, Ca^2+^, Sr^2+^, Ba^2+^ in the second coordination sphere, the percentages of the different coordinations and the distance between Gd^3+^ and the respective atoms.

Gd^3+^ Coordination Number (Percentage) Distance	MgAS3510	ZnAS3510	CaAS3510	SrAS3510	BaAS3510
Gd-NBO	4.047 (66.8%)	3.805 (68.7%)	4.209 (70.4%)	4.123 (72.6%)	4.275 (76.4%)
Gd-BO	1.979 (32.7%)	1.712 (30.9%)	1.746 (29.2%)	1.539 (27.1%)	1.310 (23.4%)
Gd-Tri	0.031 (0.5%)	0.020 (0.4%)	0.024 (0.4%)	0.017 (0.3%)	0.012 (0.2%)
Σ (Gd-O)	6.058 2.25 Å	5.538 2.25 Å	5.979 2.25 Å	5.679 2.25 Å	5.598 2.25 Å
					
Gd-Al	1.906 (16.4%) 3.51 Å	1.883 (17.0%) 3.51 Å	1.749 (14.9%) 3.57 Å	1.779 (15.5%) 3.45 Å	1.715 (14.6%) 3.57 Å
Gd-Si	5.487 (47.2%) 3.57 Å	5.177 (46.7%) 3.57 Å	5.231 (44.7%) 3.57 Å	5.008 (43.6%) 3.57 Å	4.674 (39.9%) 3.57 Å
Gd-NM	(Mg) 4.223 (36.4%) 3.33 Å	(Zn) 4.030 (36.3%) 3.27Å	(Ca) 4.732 (40.4%) 3.51 Å	(Sr) 4.703 (40.9%) 3.75 Å	(Ba) 5.329 (45.5%) 3.87Å

**Table 3:** Network modifier (NM) coordination according to molecular dynamic simulations. **Upper part of the table:** the coordination numbers (CN) and distances of the network modifying ions with oxygen in their first coordination sphere. **Lower part:** CN with Al^3+^, Si^4+^, and network modifier cations in the second coordination sphere. In the lower part, the percentages of the different cation coordinations are also given.

NM Coordination Number (Percentage) Distance	MgAS3510	ZnAS3510	CaAS3510	SrAS3510	BaAS3510
NM-O	(Mg) 4.635 2.01 Å	(Zn) 4.225 1.95 Å	(Ca) 6.230 2.37 Å	(Sr) 7.404 2.55 Å	(Ba) 9.108 2.73 Å
					
NM-Al	1.740 (18.4%) 3.21 Å	1.586 (18.7%) 3.15 Å	2.020 (16.4%) 3.21 Å	2.172 (15.6%) 3.39 Å	2.314 (15.2%) 3.51 Å
NM-Si	4.381 (46.5%) 3.27 Å	3.919 (46.1%) 3.21 Å	5.808 (47.1%) 3.57 Å	6.379 (45.8%) 3.63 Å	7.244 (47.7%) 3.57 Å
NM-NM	3.306 (35.1%) 2.97 Å	2.995 (35.2%) 2.79 Å	4.500 (36.5%) 3.39 Å	5.376 (38.6%) 3.75 Å	5.641 (37.1%) 3.99 Å

## Data Availability

Not applicable.
